# Resilience in a prehospital setting - a new focus for future research?

**DOI:** 10.1186/s13049-020-00803-z

**Published:** 2020-10-21

**Authors:** Elisabeth Jeppesen, Siri Wiig

**Affiliations:** 1 Department of Research and Development, Norwegian Air Ambulance Foundation, NO-0103 Oslo, Norway; 2 SHARE Center for Resilience in Healthcare, Faculty of Health Science, University of Stavanger, Stavanger, Norway

**Keywords:** Prehospital, Emergency, Resilience, Adaptive capacity, Framework

## Abstract

**Background:**

Handling and initiating of treatment in a prehospital setting are complex processes that involve many treatment options and include several parts of the chain of survival. Capacity to adapt to unexpected changes in the patients’ conditions or in the surroundings is a prerequisite for patient safety. Outside the healthcare sector, safety science is moving from an approach focused on the analysis and management of error (Safety I) to instead understanding the inherent properties of safety systems (Safety II). In healthcare the attention to why service providers are able to succeed under challenging conditions remains sparse. The aim of this commentary is to give a better understanding of how the concept and inclusion of resilience can inspire a new approach for future research in prehospital settings. So far, most resilience studies have been conducted in emergency departments while the role of contextual factors and adaptations in a prehospital setting has remained unexplored.

**Main body:**

In contrast to traditional research on healthcare quality and safety, which tends to focus on failures, resilience research is interested in examining the overwhelming majority of healthcare processes with successful outcomes, to determine how high-quality patient care is generated. Resilience is conceptualized as a proactive ability to adjust to potentially harmful influences and challenges rather than to resist them. To better understand and promote resilience, there is a need to explore the underlying mechanisms of adaptation, trade-offs and improvisation that occur in the emergency chain. Attention to how people respond to disruptions, challenges and opportunities is vital. There are factors, recognized and unidentified, influencing adaptation, trade-offs and improvisation. Influencing factors at different levels could be of particular value to increase knowledge to better understand resilience in a practical perspective. As prehospital work conditions are highly unpredictable and diverse, learning through everyday work could be of great value if the experiences are transferred and integrated in training and simulation.

**Conclusions:**

Empirical research is of crucial importance to build and support resilient systems and processes in a prehospital setting. We need a new framework and a new approach to how research on this topic is conducted and to support resilient performance. This should involve identifying factors that promote resilience, both on individual-, team- and system- levels.

## Background

Capacity to adapt to unexpected changes, whether these changes are in the patients’ conditions or in the environment, is a prerequisite for patient safety in a prehospital setting. Initial treatment require complex means, involve many treatment options and include several parts of the chain of survival [1], including first responder, dispatch, ambulance, advanced emergency medical treatment, triage and Emergency Departments (EDs).

Care is mostly delivered to low acuity patients, but with nonetheless complex conditions, and care is delivered to high-acuity patients with unstable vital signs in a fast-paced setting under unpredictable conditions [2]. Moreover, decisions must be made under great uncertainty. In addition, patient numbers and treatment needs, may vary considerably from situation to situation, while resources including staff, facilities and equipment often are limited. Dealing with both ordinary and extreme events, from single individuals to very complex major incidents with multiple casualties, requires adaptive capacity, flexibility, and coordination among different groups of professionals across the rescue services. Under such circumstances, human performance can be affected by individual human factors (e.g. disruptions, fatigue, and stress levels) and several environmental stressors (e.g. exposure to accidents, threatening patient behaviour) [3]. This highly dynamic work environments makes working in the prehospital environment particularly vulnerable for serious patient harm [4]. In general, the extent of medical errors and preventable harms increase under advancing complexity of care. This represents a major challenge for healthcare providers, policy makers and political leaders [5]. Outside the healthcare sector, safety science is moving from an approach focused on the analysis and management of error (Safety I) to instead understanding the inherent properties of safety systems (Safety II). In healthcare the attention to why service providers are able to succeed under challenging conditions remains sparse [6]. Due to the lack of research helping us to understand what factors may influence patient safety in a prehospital setting, the aim of this commentary is to give a better understanding of how the concept and inclusion of resilience can inspire a new approach for future research in prehospital settings. So far, most studies with a resilience perspective have been conducted in Eds [7, 8], however, generalizable models and methods for measuring resilience in EDs still remains a significant challenge, while the role of contextual factors in a prehospital setting are unexplored. We therefore need sufficient framework to be able to theorize and apply these concepts in a prehospital setting at individual, group and system levels.

## Main text

### Patient safety and resilience

There is broad agreement that a well-functioning trauma and emergency system, with a seamless treatment chain from scene to completed treatment, is essential for optimal patient outcome. However, there is a lack of studies investigating which elements contribute to increased quality and patient safety and why this treatment chain usually works well. A systematic review of studies evaluating the effects, reliability, validity and feasibility of interventions improving patient safety in emergency care identified a lack of evidence on effective safety governance strategies, particularly in the field of prehospital emergency care [9]. Simulation-based training and incident reporting systems with a focus on reducing the fear of reporting, reporting burden, and structural and systematic feedback, are promising interventions to improve the governance of patient safety in emergency care [9]. Articles discussing resilient health care in Eds recognized that to operate effectively and create value, EDs must be flexible, having the ability to rapidly adapt to the highly variable needs of patient [10]. Safety II and the resilience research is interested in examining the overwhelming majority of healthcare processes with successful outcomes to determine how high quality patient care is generated in everyday clinical practice [11]. Resilience in healthcare is conceptualized as a proactive ability to adjust to potentially harmful influences and challenges rather than to resist them, resulting in higher quality of care [12]. It’s a term that can be understood in a variety of ways, both at the individual, team and system level. Our understanding of the resilience term as a multi-level phenomenon, considers adaptive capacity to changes as a foundation for high quality care [12]. In this conceptualization resilience is defined as: the capacity to adapt to challenges and changes at different system levels, to maintain high quality care, involving flexibility, adjustments, improvisation, adaptation, and variability [12]. Current research on patient safety and resilience lacks theoretical integration of the multiple levels of the healthcare systems, from individuals and teams (micro), to organisations (meso), to regulatory bodies and policy level actors (macro) [8]. Working with severely ill or injured patients in the emergency chain demands both clinical skills and leadership at a micro level, coordination, adaptability and preparedness across tactical, operational and strategic levels at a meso level [13, 14], and development and compliance to a set of functional procedures and regulation from the regulatory bodies at the macro level [15].

### Capacity to adapt

To better understand and promote resilience, there is a need to explore the underlying mechanisms of adaptation, trade-offs and improvisation that occur in the emergency chain by exploring how people respond to disruptions, challenges and opportunities. There are factors, recognized at a personal level (self-efficacy, coping strategies and situation awareness) [16] and unidentified factors at system levels, influencing adaptation, trade-offs and improvisation. Identifying influencing factors for adaptation at different levels is of great importance for being able to conduct research and explore resilience in a practical perspective. Often healthcare performers are unable to follow procedures, checklists and standards in all situations. In some cases, adaptations can be necessary for safe service provision. Checklists can contribute to relieve stress and improve teamwork processes and often, in stressful situations, professionals sink to the level of their training and to automized procedures [17]. The professionals working in the field know that although protocols and guidelines have their place, work is only possible by continually adjusting what you do, which sometimes means improvising and working outside the “protocols”. This variability in performance is necessary to accommodate patients’ needs and unpredictable situations.

Although satisfactory individual technical skills, in combination with standardization and implementation of evidence-based guidelines, are prerequisite for developing multitasking skills, it is often not not enough to be prepared for challenging situations and better adaptive capacity. Given that the prehospital work conditions are so unpredictable and differs in scope and frequency, learning through everyday work is fundamental but not enough [18]. In a resilience perspective, learning through systematic training and simulation focusing on what works well is an under used learning source with a potential to significantly improve performance [19, 20]. Therefore, simulation can be an important tool for identifying factors that have an impact on adaptive capacity. However, apart from a small number of studies within certain clinical areas, healthcare research conducted from a resilience perspective in a prehospital setting is still in its infancy [7].

## Conclusions

Empirical research is of crucial importance to understand, build and support resilient systems and processes, including exploring and developing interventions to improve capacity for adaptive change in a prehospital setting. There is a need for new approaches and a theoretical framework to inspire future research and support resilient performance. This should seek to identify enablers of resilience, both on individual, teams and system levels. These factors are prerequisites for developing competence, exploring and understanding of work practices, for developing proactive indicators, interventions and for a deeper understanding of the complexity and developing appropriate regulations.

## Data Availability

Not applicable*.*
